# Standard-Volume Therapeutic Plasma Exchange Followed by High-Dose Continuous Renal Replacement Therapy in Patients With Hepatitis A Virus-Related Acute Liver Failure

**DOI:** 10.7759/cureus.113101

**Published:** 2026-07-21

**Authors:** Gautham Rajan, Mahesh Balakrishna, Shahid Pullat, Malik Mohammad, John Alexander, Noushif Medappil, Vinugopal S, Sarfaraz Aslam, Mohamed K, Jijo V Cherian

**Affiliations:** 1 Critical Care Medicine, Meitra Hospital, Calicut, IND; 2 Gastrointestinal Surgery, Meitra Hospital, Calicut, IND; 3 Nephrology, Meitra Hospital, Calicut, IND; 4 Gastroenterology, Meitra Hospital, Calicut, IND

**Keywords:** acute liver failure, crrt, hepatitis a, therapeutic plasma exchange, transplant-free survival

## Abstract

Acute liver failure (ALF) due to hepatitis A virus (HAV) can lead to multi-organ dysfunction with a high mortality. Extracorporeal therapies like therapeutic plasma exchange (TPE) and continuous renal replacement therapy (CRRT) often have a central role in the management of ALF. Thirteen patients admitted with HAV-related ALF between August 2024 and July 2025 were included in the study. All patients underwent standard-volume TPE (SV-TPE) followed by continuous veno-venous haemodiafiltration (CVVHDF) with an effluent dose target of 45 mL/kg/hour. Nine patients (69.2%) had hyperacute liver failure, and four (30.7%) had ALF. Seven patients met the King’s College criteria for liver transplantation. The median number of TPE sessions was one. The median duration and dose of CRRT were 76 hours and 44 mL/kg/hour, respectively. There was sustained improvement in serum bilirubin, lactate, and aspartate aminotransferase (AST) and alanine aminotransferase (ALT) levels with the offered treatment. The mean serum ammonia also reduced from 336.68 to 205.91 μmol/L after TPE, and to 123.81, 95.94, and 87.43 μmol/L after 24, 48, and 72 hours of CRRT, respectively. The mean duration of mechanical ventilation was 3.7 days. The mean ICU length of stay was 7.1 days, and the mean hospital length of stay was 13.5 days. Six patients had secondary infections. None of the patients needed liver transplantation at 21 days. The ICU-, in-hospital-, and 28-day mortality was zero; however, three patients were found to have hyperbilirubinemia at 28 days. SV-TPE followed by high-dose CRRT could significantly improve transplant-free survival in HAV-related ALF.

## Introduction

Acute liver failure (ALF) is defined as the development of jaundice, coagulopathy (prolongation of international normalised ratio (INR) >1.5), and encephalopathy in a person with no pre-existing liver disease [1]. ALF is a rare diagnosis and is often associated with dysfunction of other organs, leading to a high mortality of 26-55% [2,3].

While the predominant cause of ALF in Europe and the US is drug-induced liver injury (DILI), viral infection remains the most common cause of ALF worldwide (and especially in Asia and Africa), with hepatitis A, E, and B being the common causative viruses [3,4]. Ninety percent of ALF in India is attributable to viral hepatitis [5]. Patients with ALF should ideally be managed in an intensive care unit [6]. Early identification of patients with a likelihood of poor outcomes at baseline is vital, as a timely liver transplant may significantly improve the chances of survival [7].

Intensive supportive care has a central role in the management of ALF patients, potentially allowing the liver the time it needs to regenerate. Management focuses on maintaining hemodynamic stability and metabolic function, identifying and treating underlying causes, eliminating toxins, such as ammonia, and preventing or managing life-threatening complications, including sepsis, cerebral oedema, bleeding, and renal failure. This often involves extracorporeal therapies like therapeutic plasma exchange (TPE) and continuous renal replacement therapy (CRRT), sometimes in combination with liver assist devices [8]. While the survival rates in ALF with expectant management range between 50 and 60%, survival may improve to 80% after liver transplant [5,8]. Data from Europe suggest that ALF is the indication for 8% of all liver transplants; 19% of these cases are attributed to viral infection [8].

Kerala in Southern India saw a five-fold increase in hepatitis A cases in the year 2024, with 7967 lab-confirmed cases and 89 confirmed deaths attributed to HAV-related ALF [9]. Between August 2024 and July 2025, 43 patients were admitted to our hospital in northern Kerala with viral hepatitis A. This is a retrospective cohort study of thirteen patients who were referred to our centre for evaluation for liver transplant following hepatitis A virus (HAV)-related ALF between 01 August 2024 and 31 July 2025, but were successfully managed medically with combined TPE and high-dose CRRT and recovered even as they were awaiting liver transplant. The objective of the study was to analyse the clinical profile, details of treatment received, and the outcomes of these patients.

## Materials and methods

Study design and setting

This was a retrospective observational study conducted in a tertiary referral hospital in Kerala, India.

Inclusion and exclusion criteria

Patients who were admitted to the ICU with HAV-related ALF between 01 August 2024 and 31 July 2025, and received both TPE and CRRT, were included in the study. Patients with ALF due to aetiologies other than viral hepatitis A and those with pre-existing liver disease were excluded.

Data collection

Data were retrospectively collected from patients’ electronic medical and monitoring records after obtaining permission from institutional research and ethics committees. Demographic details, clinical data, details of treatment administered, and response to the treatment were recorded. West Haven criteria were followed for grading of hepatic encephalopathy (HE). Follow-up data were collected until 30 days after hospital discharge or until mortality, whichever happened earlier.

Methodology

All patients initially underwent TPE followed by CRRT. Patients who had not received TPE before transfer to our centre were given one session of TPE each; one patient had received two sessions of plasmapheresis prior to referral, while another patient had received four sessions. Standard-volume TPE was carried out for all patients who underwent TPE at our centre. The volume of plasma to be removed and the type and volume of replacement fluid were decided by the treating physicians. Replacement fluid used was either fresh frozen plasma (FFP) or 20% human albumin, along with crystalloids. Continuous veno-venous haemodiafiltration (CVVHDF) was carried out using the Prismaflex haemofiltration system (Baxter; Gambro Industries, Meyzieu, France) and M150 sets with polymembrane AN69 hollow fibre filters. An effluent dose of 45 mL/kg/hour was targeted. The dialysate used was Biphozyl Solution (Bieffe Medital S.P.A., Grosotto, Italy), and the replacement fluid used was Prismasol BGK 4/2.5 (Vantive US Healthcare LLC, Deerfield, IL). Three percent saline and potassium chloride were added to the dialysate and replacement solution to target serum sodium of 145-155 mEq/L and serum potassium of 4-5 mEq/L, respectively. Regional citrate was used for anticoagulation in all cases. The patients’ liver function tests, serum ammonia, serum lactate, and INR were monitored daily. CRRT was stopped when a patient's serum ammonia, serum lactate, and INR improved significantly from baseline, and there was resolution of HE.

Statistical analysis

The outcome measures monitored were normalisation of liver function tests, 21-day transplant-free survival (TFS), duration of mechanical ventilation, secondary bacterial infections, length of hospital and ICU stay, and 28-day mortality. Variables were expressed as frequencies (percentages), means, and medians as appropriate. Inferential testing was not performed due to the small sample size and absence of a control group.

Ethical approval

Approval for the study was obtained from the Institutional Human Ethics Committee, Meitra Hospital, India, before data collection (approval no.: Meitra Hospital/IEC/2025/010).

## Results

The mean age of the study population was 33.2 years (range 20-49 years). There were nine males and four females. Nine patients (69.2%) had hyperacute liver failure, and four (30.7%) had ALF. The mean APACHE II score at admission was 14.69. Seven patients met the King’s College criteria for liver transplantation for non-acetaminophen ALF at admission. Five patients had grade III HE, five had grade II encephalopathy, and three had grade I encephalopathy. Three patients had acute kidney injury at admission. Pharmacological neuroprotective and anti-encephalopathy measures, including intravenous hypertonic saline, oral lactulose, rifaximin, and supportive care like intravenous N-acetyl cysteine and intravenous vitamin K, were initiated for all patients.

The median number of TPE sessions was one (range 1-4). The mean volume of plasma removed per TPE session was 2127 mL (range 1800-2500 mL). Twenty percent human albumin, along with crystalloid, was used as replacement fluid in all patients, while nine patients additionally received FFP. The median duration of CRRT was 76 hours (mean 83.38 hours, range 48-190 hours). The median CRRT dose (rate of ultrafiltration) was 44 mL/kg/hour (mean 43.92 mL/kg/hour, range 38-48 mL/kg/hour). The clinical details of the study patients are summarised in Table 1.

**Table 1:** Clinical details of patients admitted with hepatitis A-related acute liver failure Alb, albumin; Alb FFP, both albumin and fresh frozen plasma; AKI, acute kidney injury; CRRT, continuous renal replacement therapy; FFP, fresh frozen plasma; HE, hepatic encephalopathy; ICU, intensive care unit; KCC, King's College criteria; LF, liver failure; LOS, length of stay; TPE, therapeutic plasma exchange

Clinical details of patients													
Patient	1	2	3	4	5	6	7	8	9	10	11	12	13
Age	30	41	20	38	29	42	35	35	49	24	32	32	25
Sex	F	M	F	M	M	M	M	F	M	M	M	F	M
APACHE II score	10	21	18	11	10	11	16	21	20	10	16	16	11
AKI	No	No	No	Yes	No	No	No	Yes	No	No	Yes	No	No
Grade of HE	II	III	II	I	I	II	II	III	III	III	I	II	III
Hyperacute/acute LF	Hyperacute	Hyperacute	Hyperacute	Hyperacute	Acute	Acute	Hyperacute	Hyperacute	Acute	Acute	Hyperacute	Hyperacute	Hyperacute
Met KCC	Yes	Yes	No	Yes	No	Yes	No	Yes	No	No	Yes	Yes	No
No. of TPE sessions	1	2	1	1	1	1	4	1	2	1	1	1	1
Volume of plasma removed per TPE (L)	2	2.5	1.8	2	2	2.3	2.5	2	2	2	2	1.2	2
Replacement fluid	Alb	Alb FFP	Alb	Alb	Alb	Alb FFP	Alb FFP	Alb FFP	Alb FFP	Alb FFP	Alb FFP	Alb FFP	Alb FFP
Hours of CRRT	68	76	78	50	66	76	130	190	56	48	90	68	88
CRRT dose (mL/kg/hour)	38	42	42	43	45	48	46	38	44	48	46	48	43
Days of mechanical ventilation	5	6	4	0	0	0	0	15	6	6	0	0	7
Secondary bacterial infection	No	Yes	Yes	Yes	No	No	Yes	Yes	Yes	No	No	Yes	No
ICU LOS (days)	7	8	6	5	5	6	6	18	7	4	6	7	8
Hospital LOS (days)	15	15	12	9	9	17	12	28	12	8	13	12	14

Mean serum bilirubin improved from 9.53 mg/dL prior to TPE, to 8.02 mg/dL prior to initiation of CRRT, and to 9.36, 9.64, and 9.35 mg/dL after 24, 48, and 72 hours of CRRT, respectively. This was mirrored by improvements in serum lactate, and aspartate aminotransferase (AST) and alanine aminotransferase (ALT) levels. Mean INR improved from 5.59 pre-TPE to 3.74 post-TPE; this improvement in INR was noted even in patients who did not receive FFP as replacement fluid during TPE. Mean INR further improved to 3.32, 2.87, and 2.37 after 24, 48, and 72 hours of CRRT, respectively. The mean serum lactate dropped from 4.26 mmol/L before TPE to 3.0 mmol/L after TPE and 1.85, 1.57, and 1.43 mmol/L, respectively, after 24, 48, and 72 hours of CRRT. The mean serum ammonia also reduced from 573.36 mcg/dL before TPE to 350.66 mcg/dL after TPE, and to 210.84 mcg/dL after 24 hours of CRRT, 163.38 mcg/dL after 48 hours of CRRT, and 148.9 mcg/dL after 72 hours of CRRT, respectively. The mean serum ammonia 24 hours after stopping CRRT was 150.38 mcg/dL. The temporal trend of laboratory parameters is summarised in Table 2.

**Table 2:** Temporal trend of laboratory parameters - mean values ALT, alanine aminotransferase; AST, aspartate aminotransferase; CRRT, continuous renal replacement therapy; INR, international normalized ratio; NA, not available; TPE, therapeutic plasma exchange

Parameter	Pre-TPE	Pre-CRRT	CRRT Day 1	CRRT Day 2	CRRT Day 3	Post-CRRT Day 1	At Discharge
Bilirubin (mg/dL)	9.53	8.0	9.36	9.64	9.35	9.59	13.27
AST (U/L)	5118	1373	969	631	406	308	130
ALT (U/L)	5888	2248	1793	1441	1115	746	194
Ammonia (mcg/dL)	573.36	350.66	210.84	163.38	148.9	150.38	105.14
INR	5.59	3.74	3.32	2.87	2.37	1.81	1.33
Lactate (mmol/L)	4.26	3.0	1.85	1.57	1.43	1.43	NA

Five patients needed intubation and mechanical ventilation. The mean duration of mechanical ventilation was 3.7 days (median four days, range 0-15 days). The mean ICU length of stay was 7.1 days (median six days, range 4-18 days), and the mean hospital length of stay was 13.5 days (median 12 days, range 8-28 days). Six patients had secondary infections; there were five instances of lower-respiratory tract infections and two of bloodstream infections. None of the patients needed liver transplantation at 21 days. The ICU-, in-hospital-, and 28-day mortality was zero; however, three patients were found to have hyperbilirubinemia at 28 days.

## Discussion

Only about 1% of patients with acute HAV will develop ALF [10]. Changes in immunisation patterns and improvements in standards of hygiene have led to the shift of HAV from a disease of childhood to that of young and older adults [10,11]. ALF due to HAV is more common in older patients when compared to children, and this patient group also has a poorer prognosis; it is estimated that nearly 30% of adult patients who develop HAV-related ALF either receive a liver transplant or succumb to the disease [12]. The clinical course of ALF is unpredictable, especially when hyperacute, and patients must be considered for transfer to a tertiary care unit, even if they do not currently meet criteria for liver transplant. The onset and progression of HE must be promptly recognised and managed with measures to reduce intracranial hypertension (ICH). Any patient with grade II or higher HE must be transferred to a critical care unit, and any patient with grade III or higher HE should be intubated and sedated [7,13].

ALF leads to the release of damage-associated molecular patterns (DAMPs) from injured hepatic cells and microbial pathogen-associated molecular patterns (PAMPs) in the presence of superimposed infection or bacterial translocation [14]. The DAMPs and PAMPs activate innate immune cells, which in turn produce proinflammatory cytokines (interleukin (IL)-6, IL-1ß, IL-8, tumour necrosis factor-alpha (TNF-α)) that mediate systemic inflammation and cause further hepatocellular injury. These cytokines, along with other toxins including bacterial endotoxins, bilirubin, bile acids, ammonia, and aromatic amino acids, have been proposed as important mediators in the development of both HE and multi-organ dysfunction syndrome (MODS) in ALF [15]. Levels of TNF-α and IL-6 have been shown to be significantly higher in patients with ALF [16]. An arterial ammonia level of 75 μmol/L (127.7 mcg/dL) has been identified as an important threshold below which patients with ALF rarely develop ICH [17]. On the other hand, arterial ammonia levels greater than 100 μmol/L (170 mcg/dL) at admission can predict the development of high-grade HE, and a level of 200 μmol/L (340 mcg/dL) or higher predicts ICH [17].

Therapeutic plasma exchange in acute liver failure

TPE is the extracorporeal removal of albumin-bound and water-soluble toxins from blood and replacement with plasma and/or albumin [18]. Over the last decade, TPE has become increasingly popular as a treatment option in ALF due to advances in apheresis techniques and a better understanding of its applicability in treating the pathophysiology of ALF, by providing detoxification, and modulating early immune response, while also aiding synthetic function. High-volume plasma exchange (HVPE) is plasmapheresis of 8 to 12 L or 15% of ideal body weight with FFP, and has been associated with improved TFS in ALF [14,18]. Trials have shown that patients who received HVPE had lower vasopressor requirement, serum bilirubin, and INR when compared to those receiving standard medical treatment, along with lower CLIF and SOFA scores during their ICU stay [19]. The American Society for Apheresis recommends that HVPE be considered as a first line treatment for ALF [20]. However, concerns remain regarding volume overload and the possibility of worsening of cerebral oedema following HVPE.

This has led to a shift of focus onto standard-volume plasma exchange (SVPE), which aims to replace 1.0 to 1.5 times the patient’s plasma volume with FFP or albumin. Recent studies have shown that SVPE is comparable to HVPE in improving outcomes in ALF, while being potentially safer. A significant decrease in bilirubin, INR, ammonia, pro-inflammatory cytokines, and endotoxins has been noted with SVPE, translating to improved 21-day TFS [21,22]. It is, however, important to note that TPE has not been shown to significantly reduce intracranial pressure (ICP) in ALF [14]. There is also currently no evidence-based guideline or consensus regarding the number of TPE sessions or replacement fluid that achieves the best results in patients with ALF. While Larsen et al. in their trial performed HVPE for three consecutive days, some other studies have shown improved survival even after a single session of TPE [18,21].

Role of continuous renal replacement therapy in acute liver failure

Hyperammonaemia in ALF causes cerebral oedema with increased ICH, resulting in neuronal injury and ultimately death. While initial measures to lower ICP include head elevation, sedation, and hyperosmolar therapy targeting a serum sodium of 145-155 mmol/L, CRRT has been demonstrated to effectively lower serum ammonia levels in patients with ALF [23-25]. Studies have shown that this reduction in serum ammonia level translates to reduced mortality and increased TFS at 21 days, regardless of the presence of acute kidney injury [26].

While data have been consistent regarding ammonia reduction and improved survival with CRRT in ALF, there are currently no guidelines regarding the optimal timing, technique, intensity, or duration of CRRT in ALF. A small trial has shown that there is no significant difference in ammonia clearance among the different techniques of CRRT, namely continuous veno-venous haemofiltration (CVVH), haemodialysis (CVVHD), or haemodiafiltration (CVVHDF) [27]. It has also been noted that higher rates of ultrafiltration (filtration volume of 90 mL/kg/hour vs. 35 mL/kg/hour) led to a greater reduction in ammonia concentrations and that this is possibly related to the greater dilution by the replacement fluid [24]. Reduction in ammonia concentration also correlated with greater cumulative dose of CRRT (cumulative duration of therapy hours) [25]. CRRT was also initiated early in the course of illness in both these studies [24,25]. A study in paediatric ALF patients has shown that high-dose CRRT, when initiated early, significantly increases the odds of survival [28]. This study also noted that persistently high ammonia after 48 hours of CRRT confers poor prognosis [28]. Besides ammonia, CVVHDF with a high mean effluent rate can also improve lactate clearance [29].

Hyponatremia can worsen cerebral oedema, and has been shown to independently increase mortality in patients with liver failure [30]. Sodium must be monitored and optimized as necessary during CRRT. While targeting a serum sodium of 145-155 mmol/L has been shown to decrease the incidence and severity of ICH in patients with ALF, care must be taken not to correct serum sodium by more than 8-10 mmol/L over 24 hours of treatment to reduce the risk of demyelination [31]. If the mode of dialysis used is CVVH, the target serum sodium of 145-155 mmol/L can be achieved by adding 3% saline to the replacement solution to gradually increase the concentration of sodium in the replacement solution from the initial 140 mmol/L to the desired target of 155 mmol/L [32]. In the case of CVVHDF, 3% saline must be added to both the dialysate and the replacement solution [33].

While regional citrate anticoagulation (RCA) is the preferred method of anticoagulation in critically ill patients, some concerns remain regarding its use in patients with liver failure, as impaired hepatic citrate metabolism can lead to citrate accumulation and toxicity [34-36]. As direct laboratory measurement of serum citrate concentration is not commonly available, it has been suggested that citrate toxicity in ALF patients can be diagnosed by a combination of decreased systemic ionized calcium, increased demand for calcium substitution, elevated total to ionized calcium ratio, and worsening metabolic acidosis [37]. Recent meta-analyses have also concluded that RCA is safe and effective in patients undergoing CRRT for the management of liver failure [38,39].

Combining therapeutic plasma exchange and continuous renal replacement therapy

Combining TPE with CRRT offers the theoretical benefits of both the initial quick removal of pro-inflammatory molecules, cytokines, and toxic metabolites, as well as sustained clearance of ammonia and small and medium-sized molecules from plasma, potentially allowing the liver the time it needs to recover. Data on the combined effect of TPE and CRRT are scant. A single-centre study that compared TPE combined with CRRT to TPE alone in dengue-associated ALF in paediatric patients found that combining TPE and CRRT was associated with lower mortality and significant improvements in HE, liver transaminases, coagulation profiles, and blood lactate and ammonia levels, when compared to TPE alone [40]. Another small series from a paediatric liver transplantation centre used daily TPE along with CRRT while patients were awaiting liver transplant; among the eight cases, six survived to discharge, out of which four had successful transplants, while two had spontaneous recoveries [41]. In another paediatric cohort of 23 patients with ALF or ACLF who received both CRRT and TPE, it was noted that 19 patients survived - 17 with a liver transplant and two patients with their native liver [42]. Another single-centre study, however, noted that there are no additional benefits and perhaps also potential harm when TPE was combined with CRRT when compared to CRRT alone in ALF due to aetiologies other than yellow phosphorus poisoning or Wilson's disease [43].

Limitations of the study

This is a small, single-centre study without a comparative design. Only patients with a single aetiology of ALF were studied, and there may have been potential selection and referral bias. Because the data were collected retrospectively, treatment protocols were not standardised.

## Conclusions

Our experience - though limited by small sample size and retrospective nature of the study - suggests that standard-volume TPE followed by high-dose CRRT may significantly improve TFS and could potentially serve as an effective bridge to native liver recovery in selected patients with ALF. These findings, however, are preliminary in nature, and larger prospective studies are necessary to validate these findings and to establish the safety, optimal timing, dosing, and patient selection criteria for this therapeutic approach.
